# Genetic diversity of *Avena ventricosa* populations along an ecogeographical transect in Cyprus is correlated to environmental variables

**DOI:** 10.1371/journal.pone.0193885

**Published:** 2018-03-12

**Authors:** Stella Constandinou, Nikolaos Nikoloudakis, Angelos C. Kyratzis, Andreas Katsiotis

**Affiliations:** 1 Department of Agricultural Science, Biotechnology and Food Science, Cyprus University of Technology, Athinon and Anexartisias 57, Limassol, Cyprus; 2 Department of Vegetable Crops, Agricultural Research Institute, Nicosia, Cyprus, Nicosia, Cyprus; National Cheng Kung University, TAIWAN

## Abstract

*Avena ventricosa* Balansa ex Coss. is considered the C-genome donor of the cultivated hexaploid oat and is a ‘priority’ species for conservation, since it has limited geographic distribution and the only recorded populations in Europe are present in Cyprus. The current study attempts to characterize the genetic structure and fragmentation of the species via the application of genotypic markers. It was revealed that the genetic variety was mainly allocated among the populations collected, since clustering obtained was according to the geographic origin of the samples and the habitat. Species distribution modeling showed that the most important climatic variable defining *A*. *ventricosa* distribution is the mean diurnal temperature. Furthermore, significant association of the genetic structure to environmental variables was detected; overall, a negative association to precipitation was confirmed, while significant correlations of genetic structure and the temperature at the time of anthesis and germination were established. The safeguarding of this valuable genetic resource is discussed.

## Introduction

Cyprus is the third island in size of the Mediterranean basin and is distinctive for its rich biodiversity. This is primarily due to its geographical location, unique edaphological and geological landscapes, relative isolation from continental regions, diverse climate conditions, as well as, human intervention that has uninterruptedly shaped ecological habitats since Neolithic Period [1]. Consequently, numerous ecological niches have been shaped, harboring species with diverse environmental requirements that constitute a unique mosaic of flora biodiversity. As a result, it is not surprising that more than 1900 taxonomic units have been recorded in the island of Cyprus [2].

Among the plants that constitute this great taxa wealth, primarily stand out endemic and Wild Crop Relative (WCR) species. WCR epitomize the regional adapted genotypes and constitute a genepool of desirable traits (including genes for biotic and abiotic tolerance), as well as including progenitors of cultivated species.

Cultivated hexaploid oat species (*A*. *sativa* L. and *A*. *byzantina* K. Koch, both AACCDD genome) are considered as important cereal crop globally. The preservation of their WCRs have been prioritized since 2008 via the Integrated European *in situ* Management Work Plan [3] and on top of the list is *A*. *ventricosa* Balansa ex Coss. [4]. This species was recorded to be present only in Cyprus within Europe and thus this area is considered to be an excellent candidate for *in situ* conservation. Till present, there is a complete absence of information regarding the genetic diversity and population structure of *A*. *ventricosa* in Cyprus, which is the basis for any breeding or conservation project. The need for studying the genetic diversity of *A*. *ventricosa* is also underlined by the globally low numbers of accessions kept in gene banks [5]. Underrepresentation of the species was one of the main outcomes of the global strategy for the *ex situ* conservation of *Avena* spp [6]. Furthermore, *A*. *ventricosa* has been proposed as the C-genome donor in cultivated hexaploid oat species, a fact that gives additional significance for its conservation [7].

In general, substantial correlation of genetic and ecogeographical diversity is largely expected, eventhough it does not hold true for all habitats or species [8]. A number of variables can be accounted for shaping a population’s genetic structure. For instance, biological and geographic attributes of the habitat can affect the genetic structure of plant populations. Small and remote populations are in general more vulnerable to the loss of within-population genetic variation, that reduces the viability of populations and species [9]. Furthermore, populations that have persisted over longer time of periods are anticipated to be more diverse than recent populations, since arbitrary genetic drift, accumulation of mutations, and natural selection affect their genetic structure [10]. A first step for defining a species distribution and diversity is to determine the ecogeographical boundaries. Ecogeographical data via habitat suitability modelling, also known as Species Distribution Modelling (SDM) or niche modelling, can be employed to identify suitable habitats, gaps in existing collections and to prioritize areas for collecting [11].

In the current study, we aimed firstly to reveal the genetic diversity of the *A*. *ventricosa* populations in Cyprus. For that purpose, a combination of microsatellite and AFLP markers was employed to a genepool of 63 genotypes belonging to six populations. In addition, it was intended to detect the possible genetic structure and to correlate it geographically via isolation by distance. Furthermore, it was attempted to predict the geographical dispersal of the species, using the acquired distribution and environmental data, and to identify the environmental variables that are the main contributors. Lastly, ecogeographical variety among collection sites was associated with genetic diversity indexes, in order to recognize zones and populations for *in situ/ex situ* conservation.

## Materials and methods

### Plant material, DNA extraction

**Fig 1:**
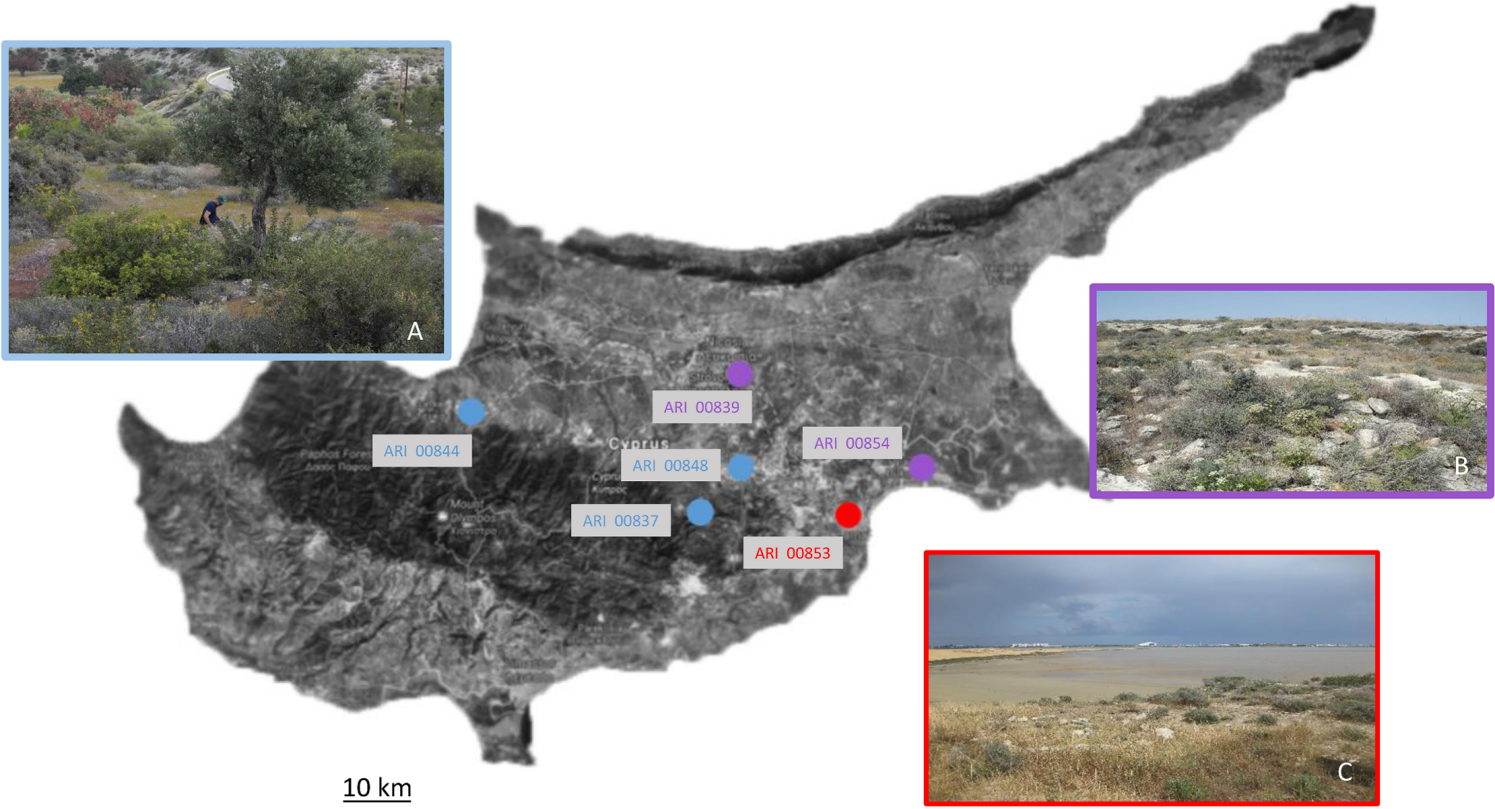
Map of Cyprus depicting the collection sites and ecological habitats of *A*. *ventricosa* populations.

Single spikelets were collected from *A*. *ventricosa* individual plant during a collecting mission. No specific permission was required for this activity, since no location was a protected area, nor the species is considered endangered/protected. The collection sites represented six different regions characterized by different edaphological and environmental conditions (**Table 1; Fig 1**). In addition, species *A*. *longiglumis* Durieu accession Cc4719 (A_s_A_s_ genome), *A*. *eriantha* accessions JIC 2087, Cc 7065, PI367381 (C_p_C_p_ genome) and *A*.*clauda* accession Cc 7044 (C_p_C_p_ genome) were also included as outliers. Seeds were germinated and total DNA was extracted from 2-week-old leaves using the Nucleospin Plant II kit (Macherey-Nagel, Düren, Germany) following the manufacturer’s instructions. The quality and quantity of DNA was defined using Nanodrop 1000 Spectrophotometer (Thermo Scientific) and confirmed in a 0.8% agarose gel electrophoresis.

**Table 1:** *Avena ventricosa* populations sampled with ecogeographical information.

Population	No of individuals used and abbreviations	Region	Ecotype	longitude	Latitude
**ARI00-837**	1p17, 1p18, 1p19, 1p20, 1p21, 1p22, 1p23, 1p24 (8)	Lithrodondas	*Pinus brutia* open forest and shrub land at the margins of Troodos mountain	33,31667	34,91666
**ARI00-839**	2p25, 2p26, 2p27, 2p28, 2p29, 2p30, 2p31, 2p32, 2p33, 2p34, 2p35 (11)	Athalassa National Park	Hot and dry area with phrygana	33,36666	35,13333
**ARI00-844**	3p3, 3p36, 3p37, 3p38, 3p39, 3p40, 3p41, 3p42, 3p43, 3p44, 3p45, 3p46, 3p47 (13)	Koutrafas	Artificial forest and shrub land at the margins of Troodos mountain	32,91666	35,06666
**ARI00-848**	4p4, 4p49, 4p50, 4p51, 4p52, 4p53, 4p54, 4p55, 4p56, 4p57, 4p58 (11)	Agios Sozomenos	Phrygana and annual grass land at the margins of cultivated land	33,41666	34,98333
**ARI00-853**	5p5, 5p59, 5p60, 5p61, 5p62, 5p64, 5p65, 5p66, 5p67, 5p68 (10)	Larnaka Salt Lake	Phrygana in the margins of salt lake	33,6	34,88333
**ARI00-854**	6p70, 6p71, 6p72, 6p73, 6p74, 6p76, 6p77, 6p78, 6p79, 6p80 (10)	Dekelia	Hot and dry area with phrygana	33,71666	34,98333

### Simple Sequence Repeats (SSR) and Amplified Fragment Length Polymorphisms (AFLPs) reactions

SSR reactions were set up in a 10 μL volume of a mixture containing 25 ng of genomic DNA, 0.75 U Taq (Promega, Madison WI, USA), 1X reaction buffer, 100 μM dNTPS and 0,1 μM of each primer **(S1 Table)** as previously described [12]. SSR markers were analyzed on an ABI 3130 Genetic Analyzer. Size standard GeneScan 500 LIZ (Applied Biosystems, Foster City, CA, USA) was included with each sample to define allele sizes. Data were analyzed using GeneMapper (Applied Biosystems, Foster City, CA, USA). AFLP reactions were carried out using the AFLP Plant Mapping kit (Applied Biosystems, Foster City, CA, USA) following the manufacturer’s instructions. EcoRI+A/MseI+C primer set was used for pre-selective reactions. Three primer pairs were used for the selective reactions, since they were found to be the most informative, after screening 32 primer combinations **(S1 Table)**. PCR products were mixed with 0.1 μL molecular size standard (GeneScan 500 LIZ, Thermo Fisher Scientific, USA) and fractioned on a Genetic Analyzer ABI Prism 3130 (Applied Biosystems). Data were analyzed using GeneMapper (Applied Biosystems, Foster City, CA, USA).

### Molecular analysis and genetic differentiation

All fragment sizes were transformed into binary data (presence/absence) and organized in a single matrix table. FreeTree [13] was employed to compute the genetic similarities between taxa and for the construction of the dendrogram, illustrated with the TreeView application (http://taxonomy.zoology.gla.ac.uk/rod/treeview.html). Popgene [14] was used to estimate Shannon diversity index and the percentage of polymorphic loci, as well as, for the construction of the population level dendrogram. Genotypic variations were calculated across various populations via the analysis of molecular variance (AMOVA) using GenAlEx 6.5 [15]. The significance of the resulting variance components and the inter-population genetic distances were tested using 999 random permutations.

Bayesian model-based clustering approach was used in order to identify the genetic structure of the *Avena ventricosa* populations, with the Structure 2.3.4 software [16]. The Structure algorithm was run using the admixture model, with 10 independent replicate runs per K value (number of clusters) ranging from 1 to 10. Each run involved a burning period of 100,000 iterations, and a post burning simulation length of 500,000. Validation of the most likely number of clusters K and graphical representations of cluster assignments was performed with pophelper (http://royfrancis.github.io/pophelper/).

### Species distribution modelling and climatic data

The initial objective was to outline the ecological niche of *A*. *ventricosa* on the Island of Cyprus. Species Distribution Modelling (SDM) can identify zones with analogous locations to those where a species has previously been detected. SDM, based on climatic information from the database of WorldClim—Global Climate Data (http://www.worldclim.org/), was performed to define *A*. *ventricosa* habitat. The climatic envelope occupied by the species is described through 19 environmental variables, listed in **S2 Table**. These information include biologically relevant temperature and precipitation layers shaped by interpolating data from weather stations around the world at a resolution of circa 1 km (30 arc-seconds), under current conditions. Furthermore, minimum, mean and maximum monthly temperature and precipitation data, were included.

The maximum entropy model, implemented in MAXENT 3.4.1 algorithm was used (http://biodiversityinformatics.amnh.org/open_source/maxent). Random null distributions were produced to assess the significance of the SDM. For this test, we built a SDM using 27 occurrence points for *A*. *ventricosa* ecotypes (**S3 Table**) retrieved from the eurisco database (https://eurisco.ipk-gatersleben.de) and the National Genebank of Cyprus (http://arinet.ari.gov.cy). The 19 Bioclim layers for current climate conditions, were used with 75% training data and a cross-validation with 25% of the remaining localities (test data) which enabled the software to compute a testing versus training data association. Specifically, an Area Under the Curve (AUC) value was calculated, providing a measure of the extrapolative power of the specified model. AUC values should exceed 0.70, in order to provide acceptable predictive power [17] after ten replications.

In order to examine the genetic structure of *A*. *ventricosa* and evaluate the effects of environmental elements on its structure, the environmental data associated with each site were extracted from all layers using Data Extraction Tool (http://dataportal-senckenberg.de/dataExtractTool/). Consequently, correspondences among population clustering, environmental factors and genetic diversity indexes were measured using Pearson’s correlation as implemented in the SPSS 22.0 software (**S4 Table**).

## Results

### Genetic diversity

**Fig 2:**
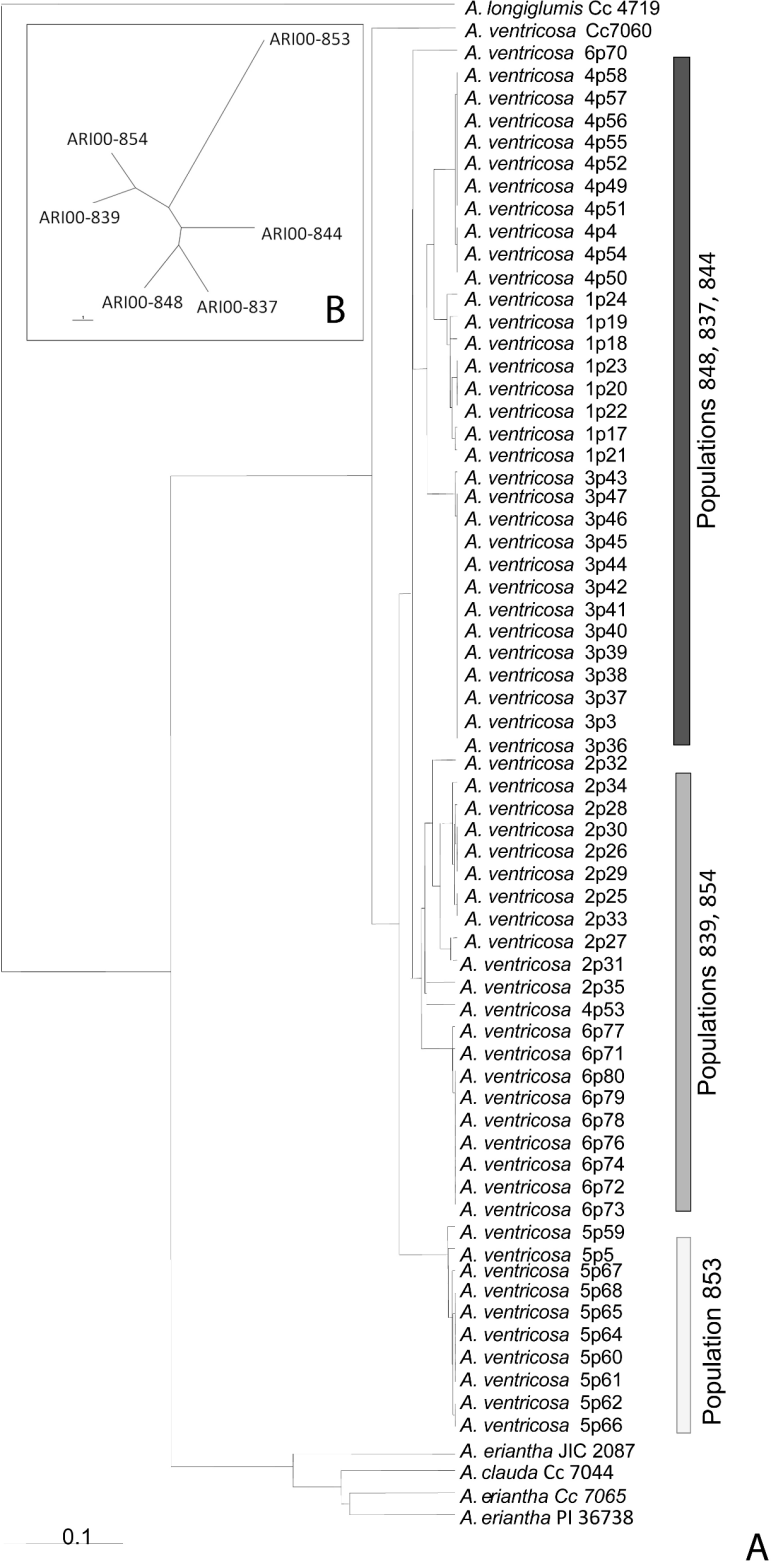
**A. UPGMA dendrogram and genetic diversity of *A*. *ventricosa* accessions.** High affinity of C_v_ sequences and differentiation from C_p_ sequences is shown. **B. UPGMA dendrogram of *A*. *ventricosa* Cypriot populations.** 183 loci of 63 individuals were grouped as six populations for analysis with POPGENE. Distance metrics among populations were based on Nei’s unbiased measure of genetic identity and genetic distance [37].

A total of 10 SSR alleles were identified in the *A*. *ventricosa* individuals, signifying the rather narrow genetic diversity detected with microsatellites. On the contrary, AFLPs generated a larger set of molecular markers producing 184 sites, with 41% of them being polymorphic. The combined data were used to calculate the genetic similarities among and within populations. Genetic relationships among the different oat genomes (C_v_, C_p_ and Α_l_) and the plant material used were depicted in the UPGMA dendrogram (**Fig 2A**). From the five diploid accessions included in the analysis as outliers, *A*. *longiglumis* Cc4719 (having an A_l_ genome) was clustered as an outgroup, while the C_p_ genome *A*. *clauda* (Cc 7044) and *A*. *eriantha* (JIC 2087, Cc 7065 and PI367381) accessions were clearly separated from the core of *A*. *ventricosa* genotypes (C_v_ genome). Furthermore, all *A*. *ventricosa* individuals were clustered according to the region collected, reflecting inter-, as opposed to, intra-population variability. The six populations sampled were organized in three distinctive clusters. Population ARI00-848 (Agios Sozomenos), ARI00-837 (Lithrodondas) and ARI00-844 (Koutrafas) formed the largest group and had genetic affinity with the second cluster composed of ARI00-839 (Athalassa National Park) and ARI00-854 (Dekelia) populations. Finally, population ARI00-853 (Larnaka Salt Lake) was the most isolated and genetically distinctive.

### Intra-population variability

In order to study the intra-population variability of the collected individuals, several indexes of descriptive statistics were calculated; among them, the number of Alleles (*Na*), the Number of Private Bands (*NPB*), the Percentage of Polymorphism (*%PP*) and the Shannon Index (*SI*) (**Table 2**). Between populations the number of loci detected was similar, ranging from 145 (population ARI00-844) to 161 loci (ARI00-854). Within each population at least one unique locus was detected; population ARI00-854 had a maximum of six. Relatively low values were calculated for the percentage of polymorphism and the Shannon index. This is however expected in strictly self-fertilized species, such as *A*. *ventricosa*. Nevertheless, populations ARI00-854 and ARI00-839 were the most polymorphic (14%) (**Table 2**). The radial dendrogram of genetic distances based on the allelic variability of the combined loci for all six populations of *A*. *ventricosa* is presented in **Fig 2B**. UPGMA analysis revealed that entries from two *A*. *ventricosa* populations formed unique clusters, while three other populations were grouped in another cluster. Both groups were interrelated with the intermediate population ARI00-853.

**Table 2:** Intra-population variability of *A*. *ventricosa* Cypriot populations.

	Ν^1^	SI^2^	Na^3^	NPB^4^	%PP^5^
**ARI00-837**	8	0.033	148	1	5.43
**ARI00-839**	11	0.067	156	5	14.13
**ARI00-844**	13	0.003	145	3	0.54
**ARI00-848**	11	0.038	155	1	8.70
**ARI00-853**	10	0.022	147	5	3.80
**ARI00-854**	10	0.052	161	6	14.13

:*Ν*^*1*^ *= Number of individuals*, SI^2^ *= Shannon Index*, Na^3^ *= Number of Alleles*, NPB^4^ *= Number of Private Bands*, %PP^5^ *= Percentage of Polymorphism*

### Analysis of Molecular Variance (AMOVA)

The clustering of *A*. *ventricosa* individuals depicted in the dendrogram, revealed that the genetic variability was mainly distributed among populations. In an attempt to allocate the genetic variability, an AMOVA was employed for the six populations. The analysis confirmed that the largest fraction of variation (85%) was between populations, while the intra-population variance was restricted to 15% (**Table 3**). The statistically significant genetic discrepancies among the Cypriot *A*. *ventricosa* populations were also demonstrated by the high *Φ*_*PT*_ value (PhiPT = 0.848, *P* = 0.001). AMOVA also permitted us to calculate the *Φ*_*PT*_ paired values among populations, summarized in **Table 3**. The comparison among pairs confirmed the genetic distinctiveness of the ARI00-853 population, that had the least genetic affinity with the ARI00-844 population (*Φ*_*PT*_ = 0.969, *P* = 0.001). This was in accordance with the previous analyses that underline the unique genetic background of that population.

**Table 3:** Analysis of Molecular Variance (AMOVA) and *Φ*_*PT*_ values for the *A*. *ventricosa* populations.

Source of Variance		Percentage Variability		PhiPT(*Φ*_*PT*_)	*P* value	
Among Populations		85%		0.848	0.001	
Within Populations		15%				
	ARI00-837	ARI00-839	ARI00-844	ARI00-848	ARI00-853	ARI00-854
**ARI00-837**	-	*0*.001*	*0*.001*	*0*.001*	*0*.001*	*0*.001*
**ARI00-839**	0.762	-	*0*.001*	*0*.001*	*0*.001*	*0*.001*
**ARI00-844**	0.893	0.852	-	*0*.001*	*0*.001*	*0*.001*
**ARI00-848**	0.739	0.749	0.909	-	*0*.001*	*0*.001*
**ARI00-853**	0.913	0.821	0.969	0.900	-	*0*.001*
**ARI00-854**	0.804	0.644	0.904	0.780	0.896	-

Lower diagonal = *Φ*_*PT*_ values,

*upper diagonal = *P* values computed with 999 permutations.

### Genetic structure

**Fig 3:**
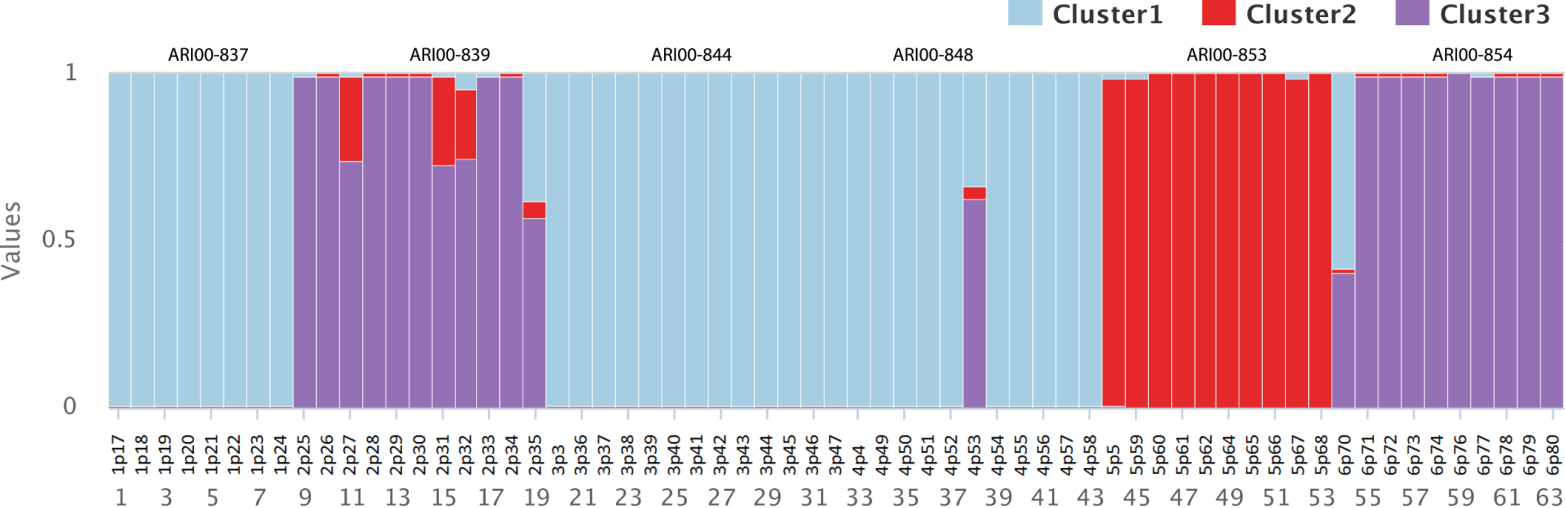
Assignment of individuals to genetic clusters resulted from the Bayesian analysis on *A*. *ventricosa* genotypes studied. The colour in each bar plot represents the probability of each individual belonging to a given genetic cluster.

In order to further elaborate the genetic structure of the populations, a Bayesian inference analysis was conducted using the Structure 2.3.4 software. We investigated the range from K = 1 to K = 10 and calculated the posterior probability for each value of K using the estimated log likelihood of K. The apparent optimal number observed was for K = 3 (**Fig 3**). As a result, the accessions used in the present study were successfully assigned into three groups (K = 3, ΔK = 135.033). In addition, a lower value at K = 8 (ΔK = 7.553) was identified. Interestingly, the majority of genotypes had a uniform genetic identity, while in few cases admixed genotypes were recorded. The Bayesian analysis revealed that populations ARI00-837, ARI00-844 and ARI00-848 were homogenous (q = 0.99), genetically affiliated and formed the largest cluster. All individuals of the ARI00-853 population were also homogenous (q = 0.99) and constituted a distinctive second cluster. Finally, genotypes of populations ARI00-854 and ARI00-839 were genetically associated shaping the third cluster. Interestingly, population ARI00-839 had a large fraction (four out of 11) of mixed genotypes (**Fig 3**).

### Species Distribution Modeling (SDM)

**Fig 4:**
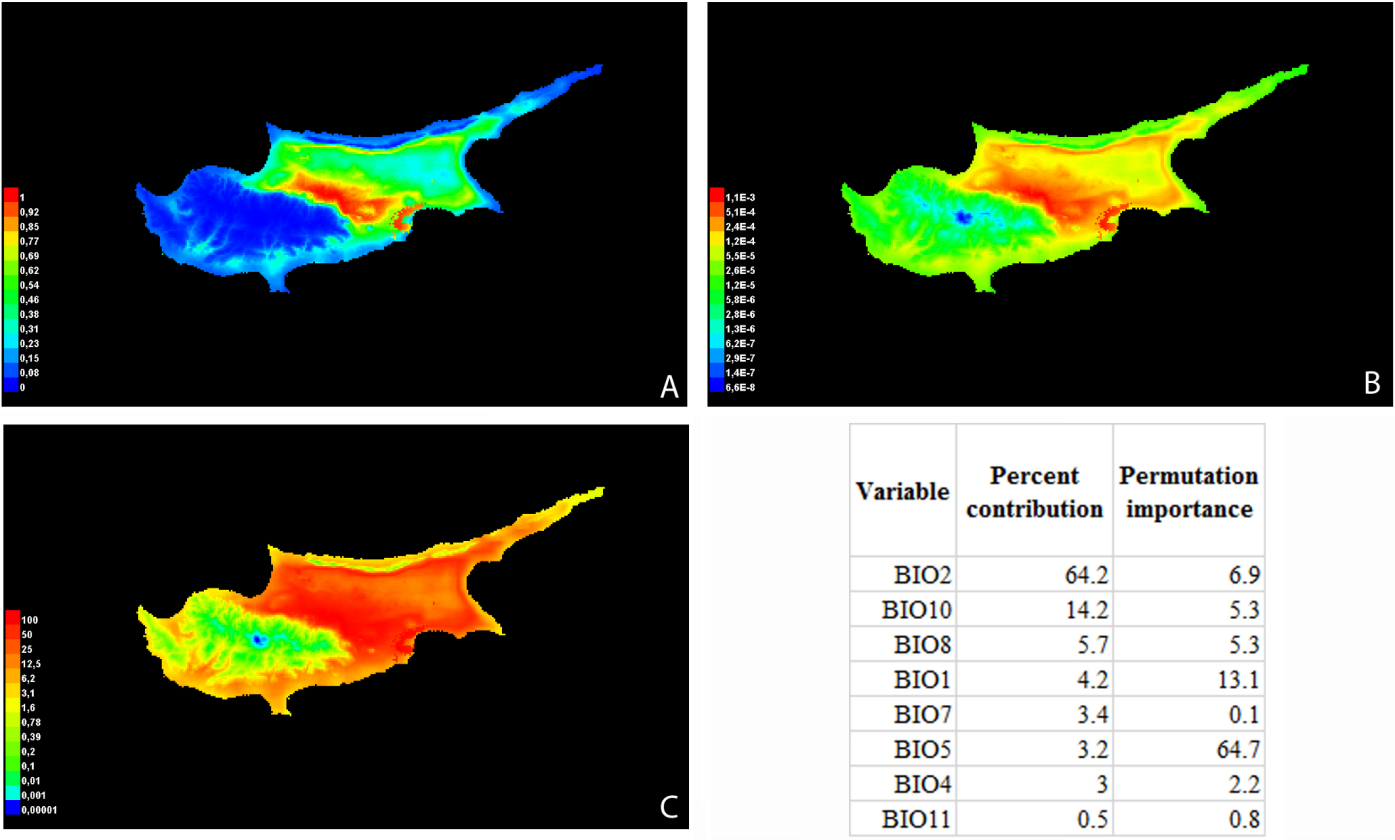
Habitat suitability for *A*. *ventricosa* determined from 19 environmental variables. Depiction based on Clogloc (A), Raw (B) and Accumulated (C) outputs. Estimates of the most important relative contributions of the environmental variables to the Maxent model are presented (values shown are averages over replicate runs).

MAXENT predictions agreed well in all models (**Fig 4**), identifying a circular band of most ideal habitat on the plains and coastal areas of Cyprus Island, and emphasizing the continental to coastal suitable habitat (warmer colors correspond to areas with a higher probability of habitat suitability for *A*. *ventricosa*). AUC values exceeded 0.70 in all analyses (0.826 ± 0.097), indicating that MAXENT models had more than acceptable predictive power. Throughout the predictions, the highest contributing climate variables were always related to mean diurnal temperature range (**Fig 4**). Taken together, the SDM analyses indicated that environmental factors clearly govern the presence of *A*. *ventricosa* in Cypriot habitats, with mean temperatures being also particularly important predictors of species presence in different areas of the island.

### Correlation with environmental factors

*Avena ventricosa* populations exhibited spatial scalability. To further understand the ecological adaptation of *A*. *ventricosa*, we analyzed the correlation of genetic diversity and population structure with the environmental factors extracted (**S4 Table**). Significant positive correlation was detected among population structure and the average temperatures in March (0.825, *P*<0.05) and in November (0.814, *P*<0.05). The Shannon index and the percentage of polymorphism were also positively correlated to mean temperature of July (0.853, *P*<0.05 and 0.837, *P*<0.05, respectively). On the other hand, a general pattern of negative correlation to monthly precipitation was detected, eventhough significant at the *P*>0.05 level.

## Discussion

The objective of the present study was to reveal the genetic variation of *A*. *ventricosa* populations in Cyprus and to further examine the existing genetic structure and its association with landscape, and climatic variation. Using a combined molecular markers approach (SSRs and AFLPs), the results of the current study show a relatively low genetic diversity percentage present at the intra-population level (in terms of SI, PP; **Table 2**). In particular, the AMOVA distributed the genetic diversity mainly among the populations (85%), supported by the high value of the Φ_PT_ index (Φ_PT_ = 0.8, p = 0.001), measuring the genetic differentiation of populations among species. Eventhough, comparison of genetic diversity estimations amongst different markers can be ambiguous, nevertheless parameters that calculate the intra- and inter-population variation, such as, the Φ_PT_ index, are generally comparable among different techniques [18,19]. Furthermore, Φ_PT_ indexes have been correlated to the fixation of homozygous alleles within subpopulations and the reproduction mode of species (cross-pollinated or self-pollinated), with self-fertile species having higher values than outcrossing species [20]. Moreover, low within-population genetic variability and low rates of heterozygosity, are additional characteristics of populations of self-pollinated species, which are in accordance to the current findings. The relatively low genetic variation of *A*. *ventricosa* populations can be attributed to the reproductive biology of the species that is a central component on how genetic variability is distributed within and among populations and defining the genomic evolution of a species [21]. Furthermore, the plant life cycle (annual vs perennial) can further reduce genetic diversity since selective pressure is not sustained for prolong periods; thus, promoting higher levels of genetic diversity [22]. Since *A*. *ventricosa* is an annual plant, the short life cycle could be an additional parameter justifying the low percentage of genetic diversity. Finally, small populations frequently encompass less within-population genetic variation related to larger populations, owed to the loss of alleles produced by inbreeding and genetic drift [23].

Nevertheless, moderate genetic differences were recorded at an inter-population level (**Table 3**), as expected for self-pollinated species [24]. In addition, SDM highlighted the environmental singularity and habitat cutoffs among *A*. *ventricosa* putative habitats in Cyprus. The distinct nature of appropriate environments corresponded to a central belt (the plain between the Troodos and Kyrenia Mountains) of the island, where the probable niche of *A*. *ventricosa* populations would be generally moderate warm and dry. The evading of rainier zones is reinforced also by earlier observations of demarcation between western and northern (wetter) zones of the island [4]. The distribution models predicted suitable *A*. *ventricosa* habitats partitioned crosswise local ecozones, rather than a simple boundary intersecting northern and western regions. For instance, Koutrafas and Lithrodondas (populations ARI00-848 and ARI00-837) are areas of relatively high altitude, at the margins of Troodos Mountain. The regional flora consists mainly of open forest and shrub land (**Fig 1**). In these areas *A*. *ventricosa* coexist with species such as *Pinus brutia* Ten., *Pistacia terebinthus* L., *Pistacia lentiscus* L., *Cistus creticus* L., *Levandula stoechas* L., *Thymbra capitata* L. and *Sarcopoterium spinosum* L. On the contrary, the regions of Dekelia and Athalassa National Park (where populations ARI00-854 and ARI00-839 were collected) are hot and dry climate territories. The most characteristic plants are *Thymbra capitata* and *Sarcopoterium spinosum*. Finally, the population from Larnaka Salt Lake (ARI00-853) was recorded and collected from phryganian type vegetation just on the margins of the lake.

The effects of environmental influences on genetic assortment are often controversial [25]. Still, the correlation of the genetic diversity within species of genus *Avena* to habitat characteristics has been mentioned in previous studies. For instance, the genetic variability of *A*. *canariensis* Baum & al. was studied by means of isoenzymes and it was determined that there were unique populations in areas with different soil properties [26]. Furthermore, it has been recorded that there was a clear discrepancy among 16 *A*. *barbata* Pott ex Link populations, in relation to climatic conditions [27].

Given the strong genetic diversity among populations between sites, we wanted to relate possible signals of significant correlation among environmental and genetic dynamics at each region. According to the SDM, the mean diurnal temperature range variables was highly essential in predicting suitable habitat for *A*. *ventricosa* populations (**Fig 4**). To quantify the significance of environmental variables imprinting to the genetic structure of *A*. *ventricosa* populations, we employed Pearson correlation tests. These established the presence of robust correlations among environmental and genetic distances among topographical populations and further suggested that climate patterns are important elements of genetic structure. Indeed, climate seems to further explain genetic variance in addition to the topography. Significant and positive correlation was accounted among the genetic structure (and diversity) of populations and the mean temperature of November and March, while a general negative correlation to precipitation was detected (**S4 Table**). Of great interest, is that the above monthly temperatures correspond to the germination and anthesis, respectively, of *A*. *ventricosa* in Cyprus, biological stages that are highly important in terms of adaptability and survival. It seems that after long-term exposure to the adverse environments, flora that grow under these conditions develop adaptive mechanisms and thus increasing sub-differentiation and genetic diversity [28]. Furthermore, significant relationships among genetic and environmental discrepancies might arise once habitat variation acts as an obstacle to gene flow, causing environmental remoteness and genetic variation, even when populations are spatially close [29], as it is the case in the present study. Though association does not ascertain causality, the main signature of adaptive evolution via time is the deterministic alteration of allelic rates between populations. Hence, the observed relationship of genetic variation to different environmental components may suggest a possible part of natural selection into shaping the divergence of *A*. *ventricosa* populations. Furthermore, a significant positive correlation of the environment (excluding the effect of geographic distance) is suggestive of the interaction of neutral dynamics and natural selection to impartial procedures of gene flow among variable population densities, as well as, genetic drift [30]. In conclusion, the environmental heterogeneity enabling adaptive deviations signifies a key constituent of natural populations today, particularly by the virtue of projected climate changes. By imposing various selection pressures crosswise geographic areas, environmental factors may alter genetic structure and distributions, but may as well, affect gene flow and local adaption [31,32]. As such, the environment represents an important aspect that ought to be progressively incorporated into evolutionary studies [33].

Natural populations have been reported to harbor great neutral genetic diversity, and in addition present considerable range of disease resistance and quantitative traits of agronomic importance [11]. Still, the number of *A*. *ventricosa* accessions from Cyprus in international collections is not adequate and are derived from a restricted number of populations [34,35]. Furthermore, regardless the spread (wide or restricted) of species, specific populations might contain significant adaptive traits, thus populations ought to be actively preserved throughout their geographical range [36]. Our portrayal of the genetic diversity allocation across the Cypriot landscape could provide a comprehensive tool for the evaluation of *in situ* conservation and further support the application of suitable sampling practices for future *ex situ* germplasm acquisitions. Based on the current study, populations ARI00-848, ARI00-839 and ARI00-853 should be monitored and maintained in the areas from which they were collected, and could further be the targets for future studies, since they present distinct genotypes, belonging to different clusters (**Fig 3**). The *ex situ* preservation of seeds, is further advised in order to maximise the possibility of retaining as many as possible distinct genotypes. Still, a more intensive special and temporal sampling would be ideal in order to get repeatable and even more robust results for a conservation plan to apply.

## Supporting information

S1 Table List of primers used and molecular markers detected in the study. (DOCX)

S2 Table Bioclimatic variables used for *A. ventricosa* species distribution models in MAXENT. Variables (http://www.worldclim.org/) are derivative from tmean, tmin, tmax and prec (average monthly mean, minimum and maximum temperature, and average monthly precipitation, correspondingly).
(DOCX)

S3 Table *A. ventricosa* coordinates in Cyprus used for the MAXENT model (data from the eurisco database/National Genebank of Cyprus). (DOCX)

S4 Table Pearson correlations of genetic structure to environmental variables. (XLSX)
