# Supplementary material for: Genetic diversity of Avena ventricosa populations along an ecogeographical transect in Cyprus is correlated to environmental variables
Source: PLoS One. 2018 Mar 12;13(3):e0193885. doi: 10.1371/journal.pone.0193885 (PMC5846772; doi:10.1371/journal.pone.0193885)
Supplement: S1 Table — (DOCX) [file pone.0193885.s001.docx]

S1 Table

List of primers used and molecular markers detected in the study

| Primer type | Marker name | Primer sequence (5'-3') | Sizes (bp) |
| --- | --- | --- | --- |
| AFLP | AFLP 1 | EcoRI-ACT / MseI-CAC | 56-494 |
| AFLP | AFLP 2 | EcoRI-ACA / MseI-CAC | 53-374 |
| AFLP | AFLP 3 | EcoRI-ACA / MseI-CAT | 53-397 |
| SSR | AM 14 | GTGGTGGGCACGGTATCA/  TGGGTGGCGAAGCGAATC | 105, 107 |
| SSR | AM 30 | TGAAGATAGCCATGAGGAAC/  GTGCAAATTGAGTTTCACG | 183, 198, 201, 204 |
| SSR | AM 31 | GCAAAGGCCATATGGTGAGAA/  CATAGGTTTGCCATTCGTGGT | 142, 145 |
| SSR | AM42 | GCTTCCCGCAAATCATCAT/ GAGTAAGCAAAGGCCAAAAAGT | 174, 177 |
