# Supplementary material for: Genetic diversity of Avena ventricosa populations along an ecogeographical transect in Cyprus is correlated to environmental variables
Source: PLoS One. 2018 Mar 12;13(3):e0193885. doi: 10.1371/journal.pone.0193885 (PMC5846772; doi:10.1371/journal.pone.0193885)
Supplement: S2 Table — Variables (http://www.worldclim.org/) are derivative from tmean, tmin, tmax and prec (average monthly mean, minimum and maximum temperature, and average monthly precipitation, correspondingly). (DOCX) [file pone.0193885.s002.docx]

S2 Table

Bioclimatic variables used for *A. ventricosa* species distribution models in MAXENT. Variables (<http://www.worldclim.org/>) are derivative from tmean, tmin, tmax and prec (average monthly mean, minimum and maximum temperature, and average monthly precipitation, correspondingly)

| Bioclimatic Code | Environmental Variable |
| --- | --- |
| BIO1 | Annual mean temperature |
| BIO2 | Mean diurnal range (Mean of monthly (maximum temperature - minimum temperature)) |
| BIO3 | Isothermality (BIO2/BIO7)(*100) |
| BIO4 | Temperature seasonality (Standard deviation of temperature * 100) |
| BIO5 | Maximum temperature of warmest month |
| BIO6 | Minimum temperature of coldest month |
| BIO7 | Temperature annual range (BIO5– BIO6) |
| BIO8 | Mean temperature of wettest quarter |
| BIO9 | Mean temperature of driest quarter |
| BIO10 | Mean temperature of warmest quarter |
| BIO11 | Mean temperature of coldest quarter |
| BIO12 | Annual precipitation |
| BIO13 | Precipitation of wettest month |
| BIO14 | Precipitation of driest month |
| BIO15 | Precipitation seasonality (Coefficient of variation) |
| BIO16 | Precipitation of wettest quarter |
| BIO17 | Precipitation of driest quarter |
| BIO18 | Precipitation of warmest quarter |
| BIO19 | Precipitation of coldest quarter |
