# Supplementary material for: Genetic diversity of Avena ventricosa populations along an ecogeographical transect in Cyprus is correlated to environmental variables
Source: PLoS One. 2018 Mar 12;13(3):e0193885. doi: 10.1371/journal.pone.0193885 (PMC5846772; doi:10.1371/journal.pone.0193885)
Supplement: S3 Table — (DOCX) [file pone.0193885.s003.docx]

S3 Table

*A. ventricosa* coordinates in Cyprus used for the MAXENT model (data from the eurisco database/National Genebank of Cyprus)

| Species | Longitude | Latitude | Population |
| --- | --- | --- | --- |
| *Avena_ventricosa* | 32,88444444 | 35,08138889 |  |
| *Avena_ventricosa* | 32,94277778 | 35,07666667 | ARI00-844 |
| *Avena_ventricosa* | 32,9725 | 35,09916667 |  |
| *Avena_ventricosa* | 33,1275 | 35,07027778 |  |
| *Avena_ventricosa* | 33,13611111 | 35,16388889 |  |
| *Avena_ventricosa* | 33,25916667 | 34,79305556 |  |
| *Avena_ventricosa* | 33,27388889 | 34,70666667 |  |
| *Avena_ventricosa* | 33,30972222 | 34,9875 |  |
| *Avena_ventricosa* | 33,34222222 | 34,94333333 | ARI00-837 |
| *Avena_ventricosa* | 33,35972222 | 34,95833333 |  |
| *Avena_ventricosa* | 33,3675 | 34,9525 |  |
| *Avena_ventricosa* | 33,39277778 | 35,13472222 | ARI00-839 |
| *Avena_ventricosa* | 33,39722222 | 34,86833333 |  |
| *Avena_ventricosa* | 33,40277778 | 35,14777778 |  |
| *Avena_ventricosa* | 33,41055556 | 35,13944444 |  |
| *Avena_ventricosa* | 33,42888889 | 34,995 | ARI00-848 |
| *Avena_ventricosa* | 33,43555556 | 35,0675 |  |
| *Avena_ventricosa* | 33,43805556 | 35,07694444 |  |
| *Avena_ventricosa* | 33,44277778 | 34,93944444 |  |
| *Avena_ventricosa* | 33,44333333 | 34,93972222 |  |
| *Avena_ventricosa* | 33,4875 | 34,95277778 |  |
| *Avena_ventricosa* | 33,5675 | 34,94361111 |  |
| *Avena_ventricosa* | 33,60916667 | 34,89527778 | ARI00-853 |
| *Avena_ventricosa* | 33,725 | 35,01 | ARI00-854 |
| *Avena_ventricosa* | 33,76722222 | 34,99333333 |  |
| *Avena_ventricosa* | 34,06777778 | 34,96638889 |  |
| *Avena_ventricosa* | 34,07666667 | 34,96717778 |  |
